# Sustainable development of protein-fortified Quince *(Cydonia oblonga)* fruit leather using almond powder: Nutritional quality, antioxidant capacity, and tensile strength

**DOI:** 10.1371/journal.pone.0348789

**Published:** 2026-07-10

**Authors:** Nagina Batool, Zunaira Arshad, Nabeel Ashraf, Farah Jameel, Rola A. Jalloun, Alanood A. Alfaleh, Duaa Altuwairki, Abeer A. Aljehani, Mahmoud Helal, Awatif Almehmadi, Rokayya Sami

**Affiliations:** 1 National Institute of Food Science and Technology University of Agriculture, Faisalabad, Pakistan; 2 Department of Clinical Nutrition, Taibah University, Universities Road, KSA, Medina, Saudi Arabia; 3 Department of Food and Nutrition, Faculty of Human Sciences and Design, King Abdulaziz University, Jeddah, Saudi Arabia; 4 Department of Mechanical Engineering, Faculty of Engineering, Taif University, Taif, Saudi Arabia; 5 Department of Clinical Nutrition, Faculty of Applied Medical Sciences, Umm AL-Qura University, Makkah, Saudi Arabia; 6 Department of Food Science and Nutrition, College of Sciences, Taif University, Taif, Saudi Arabia; Amity University Noida, INDIA

## Abstract

Fruit leathers are delicious, convenient and shelf-stable alternative of fresh fruits offering benefits of valuable sources of nutrients, dietary fiber and bioactive compounds. The novelty of almond-fortified quince fruit leather lies in combining quince’s natural antioxidants and dietary fiber with the protein and healthy nutrients of almonds to produce nutritionally enriched, plant-based snack. This fortification improves the nutritional and functional value compared to conventional carbohydrate-rich fruit leathers. This research was done to assess the impact of adding almond powder as a protein fortifier in the quince fruit leather. Fruit leather samples were prepared by hot air drying at 65 °C for 8–12 hours. There were five formulations (T_0_; control; 100% quince pulp), T_1_ (95% quince pulp + 5% almond powder), T_2_ (90% quince pulp + 10% almond powder), T_3_ (85% quince pulp + 15% almond powder) and T_4_ (80% quince pulp + 20% almond powder). The moisture content and nitrogen-free extract (NFE) reduced significantly among treatments (T_0_-T_4_) with values of 23.22% −11.24% and 66.96% −15.79%, respectively. While, fat, fiber, protein and ash contents were significantly increased all over the treatments, with values recorded at T_0_-T_4_ (6.60%−33.32%), (17.30%−32.72%), (0.63%−9.18%), and (3.53%−13.19%), respectively. The total phenolic content (TPC), total flavonoid content (TFC), and DPPH radical scavenging activity were also increased significantly and ranged between 164.75 to 170.32 mg GAE/g, 82.91 to 90.38 mg QE/g and 58.29 to 71.53 mg TE/g in T_0_ and T_4_, respectively. Water and oil absorption rates were reduced (T_0_-T_4_) from 2.17 to 2.12 mg/g and 2.20 to 0.60 mg/g, respectively. The amount of vitamin C dropped considerably in T_0_ (17.43 mg/100g) to T_4_ (7.67 mg/100g), respectively. The addition of almond powder significantly affected the texture of quince fruit leather, with tensile strength decreasing from 1.46 MPa (T_0_) to 0.04 MPa (T_4_), elongation from 15.66% to 1.07%, and rupture strength from 64.93 N to 22.88 N, reflecting disruption of the pectin network and increased porosity caused by dispersed almond particles. Browning index rose substantially when almond powder is added, and it varies between 33.12 and 51.19 among T_0_-T_4_ samples. This study indicated that the T_2_ showed the best results and demonstrated high nutritional and tensile strength. This fruit leather could potentially be regarded as a high-nutrient, healthy and convenient alternative snack food.

## Introduction

Quince (*Cydonia oblonga Mill*.) is a shrub of the family of Rosaceae. It has hard flesh and astringent bitter flavor, therefore the consumption of this food in raw is restricted, so it is usually processed into other food forms like jam, jelly, and dried slices [1]. Quince has been grown in Iran, Turkey and northern regions of Pakistan (Gilgit Baltistan). In Urdu, bahi and in Balti, it is called shadool. Quince is a poorly used fruit in Pakistan, although its nutritional value and economic and medical capacity are high [2].The fruit is described as having a bright golden-yellow hue, hard texture, and acidic taste, and even though they do not consume it fresh because of their astringency, it is full of functional and bioactive compounds [3]. Quince fruits are also consumed to make jam, jelly, juices and puree and marmalades. Some studies have indicated that quince has high number of bioactive components in the form of phenols, flavonoids, polysaccharides, dietary fiber, and organic acids which play different health promoting roles. As a result, the nutritional deficiency can be overcome by using quince in value-added products, which would aid in enhancing dietary fiber and polyphenols intakes [4]. Almond (*Prunus dulcis*) is a tree, which is very variable in size, form and mode of growth. Almonds are highly nutritionally balanced foods and super source of lipids, carbohydrates, monounsaturated fatty acid, dietary fiber, vitamin E, riboflavin as well as various essential minerals. The use of almonds as a healthy food is highly encouraged and the recommended dosage of almonds is 30-50g/day. Regular intake of almonds has been linked to a wide range of health advantages, especially in the number of cardiometabolic disorders, obesity, high blood pressure, and diabetes mellitus [5]. Fortification is the process of adding nutrients to foodstuffs to improve the nutritional quality and counter nutrient deficiency. Modern fortification practices are moving toward the use of functional ingredients that include spirulina, moringa, flax seeds among others to enhance health attributes. Consequently, fortified fruit products would offer better nutrient content, satiety, and better health outcomes of populations [6]. Fruit leather is a snack that is dried, brawny, and gummy as it is made of fruit pulp, fruit juice, or fruit puree with or without sweeteners and natural additives [7]. It is made by removing the moisture content of fruit pulp through drying methods as hot-air ovens or dehydrators, producing a thin shelf-stable sheet. It is also possible to make fruit leather using concentrated fruit juice, but adding other food ingredients like sweeteners and preservatives [8]. Drying is regarded as one of the effective preservation mechanisms since it is not very expensive, and requires less packaging, storage and transportation. One of the most popular methods of preserving fruits at the industrial level is conventional drying. However, in economical ways, traditional sun drying is dangerous because it can be contaminated, thus more sophisticated drying techniques of microwave, infrared, vacuum, freeze drying, and oven drying are used. Oven drying is the most commonly used among them because it has the effectiveness in removing moisture, extending shelf-life and preserving flavors and nutrients. Fruit leather weighs little, can be carried around and it does not need refrigeration thus it is a light snack to take on a trip, on a hike and even at school. Previous studied reported that fruit leather is prepared by using apple pulp and fortified with Fig which is a good source of calcium, iron, vitamins, minerals, and antioxidants. This fortified leather can be used to maintain the bones health [9]. The originality of the almond fortified quince fruit leather consists in the fact that the natural antioxidants and dietary fiber present in quince are added to the protein and nutrient-containing almonds to create a nutritionally fortified and plant-based snack. This will enhance the nutritional balance and functionality over the traditional fruit leathers that contain carbohydrates in large quantities. Thus, the main objective of this research is to develop and characterize almond powder fortified quince fruit leather and evaluate the nutritional, physicochemical and, sensory properties of quince fruit leather.

The novelty of the study is that the Quince (*Cydonia oblonga*) is an underutilized fruit known for its high pectin content, considerable antioxidant potential, and unique flavor profile, which make it a suitable candidate for functional food applications. The use of almond powder as a fortifying ingredient offers a novel strategy, as it enhances protein content while supplying beneficial lipids and essential micronutrients. Moreover, the combination of quince and almond is anticipated to produce a synergistic effect, improving the physicochemical characteristics, texture, and sensory quality of the final product. Hence, this study focuses on the development and evaluation of almond-fortified quince fruit leather, aiming to contribute to value addition, nutritional improvement, and the advancement of commercially viable functional snack products.

## Materials and methods

### Raw materials selection

Fresh, fully ripe and bright-yellow quince fruits and almond powder were bought in Skardu. The quince fruit leather preparation was done in the labs of National Institute of Food Science and Technology at University of Agriculture, Faisalabad. All the chemicals and reagents applied were analytical quality reagents and were bought at Merck, Germany.

### Leather preparation

The quince fruits were washed using a lot of running water to get rid of dusts and surface dirt. The peeling, deseeding, slicing and cooking of the fruits under clean water (10–15 min) followed to prepare the enzymes inactive and the tissue soften. The method used to prepare pulp was that of [10].To make protein-enriched quince fruit leather, quince pulp and almond powder were mixed in various ratios as revealed in Table 1. As natural sweetener, Stevia was utilized. The ready pulp was put in a uniform layer on trays that were covered with stainless steel that was lightly oiled. The pulp layer was dried at a temperature of 65 °C in a dry oven in 8–12 hours and kept the thickness of the pulp layer at 3 mm. The trays were then dried and once they were completely dry, they were cooled and then the fruit leather was cut into strips and packed in polyethylene bags. The samples that were prepared were kept at 4 °C for further analysis analyses.

**Table 1:** Treatment plan for protein fortified quince fruit leather preparation.

Treatments	Quince pulp (%)	Almond powder (%)
**T**_**0**_	100	---
**T**_**1**_	95	5
**T**_**2**_	90	10
**T**_**3**_	85	15
**T**_**4**_	80	20

### Proximate analysis of protein fortified quince leather

Sample quince fruit leather fortified with protein was examined in terms of proximate composition such as moisture, crude fat, crude fiber, ash, crude protein, and nitrogen-free extract (NFE) by following the given procedure in literature [11].

#### Moisture contents.

Moisture contents of all treatments of protein fortified quince fruit leather were estimated by placing the 5g sample in a hot air oven at 105 °C for 24 hours. The moisture contents of each treatment were calculated by this formula:


$$\begin{array}{c} Moisture(\%)=\frac{Weight\;of\;fresh\;sample\;-\;Weight\;of\;dry\;sample\;}{Weight\;of\;fresh\;sample}\;\times \text{1}\text{0}\text{0} \end{array}$$

#### Crude fat contents.

The Soxhlet apparatus was used to determined crude fat content of fruit leather. Dry samples were put in thimbles and washed with n-hexane six times using a siphon. Thimbles were dried using a hot air oven (1–2 hours) and then weighed. The percentage of fat was obtained by calculating the following formula:


$$\begin{array}{c} Crude\;fat\;(\%)=\frac{Weight\;of\;extracted\;fat\;}{Weight\;of\;sample\;}\;\times \text{1}\text{0}\text{0} \end{array}$$

#### Crude fiber contents.

To determined crude fiber content, fat free sample were used. Fat free form were treated with sulphuric acid (1.25% H2SO4) and alkali (1.25% NaOH) to eliminate soluble compounds. The residues were dried at 100 °C at 1 hour, charred, and finally ash at a muffle furnace at 500 °C at 2 hours until grey. After that weight the sample again, and the crude fiber content was determined by using this formula:


$$\begin{array}{c} Crude\;fiber\;(\%)=\frac{Weight\;of\;residue\;after\;digestion\;}{Weight\;of\;sample\;}\;\times \text{1}\text{0}\text{0} \end{array}$$

#### Crude ash contents.

Ash concentration was determined by incinerating the 5g of sample in a crucible until it was smoke-free after which it was subjected to muffle furnace at 550–600 °C and 6 hours. The samples were converted into greyish white ash. After cooling the sample, they were weighed, and crude ash was obtained by using this formula:


$$\begin{array}{c} Ash\;(\%)=\frac{Weight\;of\;fresh\;sample\;}{Weight\;of\;ash}\;\times \text{1}\text{0}\text{0} \end{array}$$

#### Crude protein contents.

The kjeldhal method that involved digesting, distillation, and titration was used to determine the protein content. The procedure of digestion was the heating of 2 g of sample with H_2_SO_4_ and catalyst until a pale green solution was acquired. Released ammonia was distilled into 4% boric acid and titrated with 0.1 N H_2_SO_4_ in the presence of methyl red as an indicator. Nitrogen content was calculated by following formula:


$$\begin{array}{c} Nitrogen\;(\%)=\frac{(Vb\;-\;Vs)\times N\;\times \text{1}\text{4}\;\times \text{1}\text{0}\text{0}\;}{Weight\;of\;sample\;}\;\times \text{1}\text{0}\text{0} \end{array}$$

Where,

Vb = volume of standard acid used for blank (mL)

Vs = volume of standard acid used for sample (mL)

N = normality of acid

14 = atomic weight of nitrogen

To calculate the protein content, Nitrogen (%) × 6.25.

#### Nitrogen free extract (NFE) contents.

Nitrogen free extract (NFE) in a protein fortified quince leather sample was evaluated by using the following expression.


$$\begin{array}{c} \text{NFE }(\%)\;=\;(\text{100 }-\text{ moisture }\%\;+\text{ crude protein }\%+\text{crude fat }\%+\text{ crude fiber }\%\;+\text{ash }\%) \end{array}$$

### Physicochemical analysis of protein fortified quince leather

#### Total acidity.

Total acidity of quince fruit leather was determined following the protocol given in [12]. A 5g of quince leather was dissolved in 50 mL of distilled water. From this, 10 mL sample was taken, 2–3 drops of phenolphthalein added, and titrated with 0.1N NaOH until a pink endpoint appeared. The volume of NaOH used was recorded. Total acidity of fruit leather was calculated by using this formula:


$$\begin{array}{c} (\text{Total Acidity }(\%)=\text{0}.\text{0064}\frac{\text{0006}.\;\times \text{Vol}.\text{ of NaOH used}(\text{ml})\;}{weight\;of\;sample}\times \text{1}\text{0}\text{0} \end{array}$$

#### Total soluble solids (ºBrix).

The TSS of fruit leather was determined by using a digital refractometer (Erma, Japan) [12].

#### pH.

pH of fruit leather was estimated by using a pH meter (HANNA instruments, Model, 23044) [11].

### Phytochemical analysis

To determined phytochemical profile of fruit leather, 5g of sample was mixed with 50 mL of 80% ethanol, and kept at room temperature for 12 hours. The mixture was centrifuged at 13,500 rpm for 30 min, and supernatant was collected as the extract. Store the sample in air tight bottle for further analysis [13].

#### Total phenolic content (TPC).

TPC of fruit leather was determined by using a spectrophotometer [13]. 0.5 mL extract was mixed with 2.3 mL of 10% Folin-Ciocalteu reagent and 2.5 mL of 7.5% Na₂CO₃. Incubated at 45 °C for 45 minutes and absorbance was measured at 765 nm using a spectrophotometer. Gallic acid was used as standard to calculate the TPC.

#### Total flavonoid content (TFC).

TFC of fruit leather was estimated by the described method given in [14]. 0.5 mL extract was mixed with 2.5 mL distilled water, 0.15 mL 5% NaNO₂, 0.3 mL 10% AlCl₃, and 1 mL NaOH in a test tube, with gentle shaking. Absorbance was measured at 510 nm. The catechin was used as a standard to calculate the TFC.

### Antioxidant activity

The antioxidant activity of fruit leather was estimated using the DPPH (2,2-diphenyl-1-picrylhydrazyl) assay by following the method of [15]. 50 µL of extract was combined with 3 mL of a 0.004% (v/v) DPPH methanolic solution. After 30 min, the absorbance was measured at 517 nm using a spectrophotometer. The reduction in DPPH radical by the sample was calculated by the formula:


$$\begin{array}{c} \left(\text{DPPH radical scavenging activity}(\%)\;=\text{ 100 }\times \;({\text{A}}_{\text{0}}\;-\;{\text{A}}_{\text{1}}/{\text{A}}_{\text{0}}\right) \end{array}$$

Where,

A_0_= absorbance of control

A_1_= absorbance of quince leather.

### Vitamin C

Vitamin C content was determined by the described method in [16]. 10g sample was blended with 50 mL of 5% metaphosphoric and 10% acetic acid, transferred to a 100 mL flask, and centrifuged at 1350 rpm for 10 minutes. 1 mL supernatant was treated with 3% bromine solution, followed by 10% thiourea to neutralize excess bromine. Then 1 mL of 2,4-dinitrophenylhydrazine was added. The solution was incubated at 37 °C for 3 hours, cooled, and reacted with 85% H₂SO₄. Absorbance was measured at 521 nm.

### Color analysis

The values of color parameters (L*, a*, b*) were determined with the help of a calibrated colorimeter, according to method describe by [11].

### Mineral analysis

Mineral analysis of fruit leather was carried out according to the given method described in [17]. The sample was weighed in a crucible, burned until smoke-free, and ashed at 550 °C in a muffle furnace. The ash was dissolved in 2 mL HNO₃, transferred to a 100 mL flask, and mixed with 2.5 mL strontium solution. Mineral content was determined using a spectrophotometer.

### Texture analysis

Texture analysis of fruit leather was performed by using the TX-700 texture analyzer by following the method described in [18]. The experiment was carried out at room temperature of 20 ºC. By performed this test the determined parameters were hardness, springiness, cohesiveness, gumminess, and chewiness.

### Tensile analysis

TST-01 Tensile Tester was used to determine the tensile strength of quince fruit leather. Tensile strength test was applied on 20 mm length and 4 mm width of quince fruit leather for 10 mm/min. After that, the elongation (%), rupture strength (N), and tensile strength (N) were determined by using method described in [19].

### Oil absorption capacity (OAC)

The oil absorption capacity of fruit leather was determined according to given method [11]. 10 mL oil (V) was mixed with 1g sample in a 25 mL centrifuge tube and centrifuged at 4000 rpm for 20 minutes. Separated, and measured the supernatant oil volume (V₂). The oil absorption capacity of fruit leather was determined by using this formula:


$$\begin{array}{c} \text{OAC }(\text{g}/\text{ml})\;=\;{\text{V}}_{\text{1}}-{\text{V}}_{\text{2}} \end{array}$$

Where,

V_1_ = Total volume of oil used

V_2=_ Supernatant oil volume

### Water absorption capacity (WAC)

The water absorption capacity of fruit leather was determined according to given method [11]. 10 mL of water (V_1_) was placed into a 25 mL centrifuge tube. Added 1 g of powdered sample into centrifuge tube. Centrifuged the sample for 20 minutes at 4000 rpm. The supernatant oil was separated and measured the volume (V_2_).

### Sensory evaluation

The sensory evaluation of fruit leather was done by trained 20 panelists and their age between 24–50 years from the University of Agriculture, Faisalabad using a 9-point hedonic scale (9= like extremely to 1 = dislike extremely) [16]. The assessment was conducted in a well-lit, odor-free environment under controlled conditions to reduce external interference. Samples were coded and presented in a randomized sequence to eliminate bias. The sensorial attributes including color, flavor, appearance, texture and overall acceptability were evaluated.

### Statistical analysis

The obtained data were subjected to statistically examination to determine the level of significance as described by [20], using statistic 8.1. The data were in triplicates and results were expressed in mean and standard deviation using a one way ANOVA followed by Tukey’s post-hoc test to determine significant differences (p < 0.05) among means.

## Results and discussion

Table 2 gives the average proximate composition of almond powder. The direct chemical analysis showed that it was composed of moisture (4.33%), fat (52.8%), crude fiber (10.1%), protein (21.57%), and nitrogen-free extract (NFE) (9.27%). Such findings correspond to the findings of [21, 22].

**Table 2:** Proximate composition of almond powder.

Components	Almond powder (%)
Moisture	4.33 ± 0.28
Fat	52.8 ± 0.72
Fiber	10.1 ± 1.12
Protein	21.57 ± 0.67
Ash	3.13 ± 0.42
NFE	9.27 ± 0.25

### Proximate analysis of protein fortified quince leather

#### Moisture contents (%).

**Fig 1:**
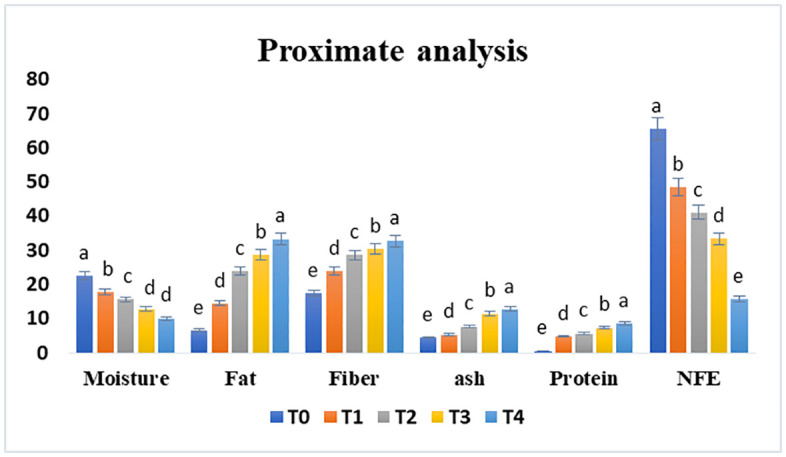
Influence of concentration of almond powder on proximate of quince leather.

Moisture content of protein-fortified quince fruit leather is shown on Fig 1. It was observed that the addition of almond powder significantly decreased the moisture contents of quince fruit leather. T_4_ showed the lowest moisture content (10.11%) as compared to T_0_ (22.613%), it was due to the hydrophilic nature of quince pulp and hydrophobic nature of almond powder, which caused water loss during dehydration. Similar tendencies have also been reported for iron-enriched jamun leather [23].It was observed that desi jamun had a higher moisture content (84.08 ± 0.03) compared to ra jamun (82.11 ± 0.04). Another study in which mango pulp fortified with natal plum at different ratios 5:1, 3:1 and 2:1. It was observed that the moisture content was increased with decreased ration of natal plum and the treatment of 5:1 had the lowest moisture content [24].

#### Crude fat contents (%).

The crude fat content was significantly increased by the addition of almond powder (Fig 1). The incorporation of almond caused increasing trend of fat contents in quince fruit leathers because almonds are rich in lipids in nature and have high levels of mono-unsaturated and poly-unsaturated fatty acids [25].T_4_ showed the highest fat content with 33.32% in contrast to T_0_ which showed the lowest value with 6.60%. Similar increases in fat content have been reported for biscuits made from buckwheat and almond flour [26]. In another study, fruit leather was prepared by banana fruit enriched with encapsulated flaxseed oil. The observed fat contents in leather increased up to 3.27% due to addition of flaxseed oil [27]. Another study followed a same trend to this study, where mango fruit leather was fortified with red dragon fruit peel to enhance the fiber content. It was observed that the treatment containing red dragon fruit feel had a higher fat content (0.54 ± 0.03) compared to treatment without red dragon fruit peel (0.52 ± 0.04) [7].

#### Crude fiber contents (%).

The fiber contents of quince fruit leathers were determined and results are shown in Fig 1. The addition of almond powder increased the fiber contents of quince fruit leather because almond is a rich source of cellulose and hemicellulose which are the structural polysaccharides of cell wall which work as a dietary fiber and normally 12.5g/100g fiber present in almond [28]. In this study, T_4_ showed highest fiber contents (32.72%) as compared to T_0_ (17.303%), it was due to the high dietary fiber content of almond, which demonstrated a synergistic effect with pulp and enhanced over all fiber content of quince leather. The same trend was observed in a study where fruit leather was prepared from different fruits (consisting pear, red currant, peach and haskap berry) fortified with fructooligosaccharides (FOS) from chicory and Jerusalem artichoke. The result observed was increasing trend in crude fiber 8.33 ± 0.267.40 ± 0.04% [29]. Similarly, comparable results were reported where mixed vegetable-fruit leather was prepared with various portion of mustard greens. The treatment had the ration of mustard greens 80%: fruit 20% had lowest crude fiber (0.68 ± 0.07) compared to the treatment had mustard greens 100%: fruits 0% had highest crude fiber (0.83 ± 0.24) [30].

#### Ash contents.

The ash contents of quince fruit leathers were determined and results are shown in Fig 1. The incorporation of almond powder increased the ash contents of quince fruit leather because almond contained a good amount of minerals which ultimately increased the ash contents. T_4_ showed highest fiber contents (12.763%) as compared to T_0_ (4.443%) because almond consist high mineral contents, which enhance the total inorganic matter in the quince leather. Similar results were obtained from [31] where papaya leather was prepared by fortifying soy slurry. The obtained result was that the control treatment had lowest ash content (0.56 ± 0.03) as compared 70% soy slurry contained treatment (2.08 ± 0.24). Similarly, comparable results were reported where an increase in ash contents of cookies were observed with increasing almond the powder concentration from 0–20% and the obtained results of ash contents were 1.62–2.72% [32].

#### Crude Protein.

The crude protein content was increased significantly by increasing levels of almond powder (Fig 1). Almonds are rich in essential amino acids like lysine, leucine, and arginine, making them a great plant-based protein source [33]. T_4_ showed highest fiber contents (8.593%) as compared to T_0_ (0.56%) because the amino acids present in almonds were involved to enhance the protein contents in quince leather. Similar results were obtained from the study in which crackers were prepared by using wheat flour and almond flour in different ratios and the treatment which contained high almond powder showed higher protein contents of 12.00–21.47% [34]. Another study showed similar results where walnut, almond, pumpkin seed, chia seeds, oats and soy milk were used to prepare a blend in different concentration, the blend with almond showed highest level of protein contents 26–31% [35]. The same trend was observed where red dragon fruit and watermelon rind were enriched with seaweed in varying ratios (0%, 10%, 20%, and 30%). It was observed that the treatment had 30% seaweed had highest protein content 1.66 ± 0.07 compared to control treatment 1.27 ± 0.03 [36].

#### Nitrogen free extract (NFE).

The NFE contents of quince fruit leathers were analyzed and results are shown in Fig 1. The addition of almond powder in quince fruit pulp decreased the overall NFE contents of quince fruit leather. In this study T_4_ showed lowest fiber contents (15.846%) as compared to T_0_ (65.503%) because almond contained high amount of fat, fiber and protein so it alternatively affected the NFE contents. The same trend observed in a study where almond powder and carrot flour blends were prepared and the observed results also showed decreasing effect on NFE contents and the range was 81.18–71.27% [32]. Another study showed similar results where flaxseed was fortified in banana pulp to prepared functional fruit leather and it was observed that the NFE contents was significantly reduced [27].

### Mineral analysis

Table 3 shows the mineral content of quince fruit leather that is fortified with almonds. The addition of almond powder significantly increased the mineral contents (Mg, Zn, Fe and Ca) in quince fruit leather. In this study, a gradually increased was observed in magnesium (from 6.66 mg/100g in T_0_ to 60.7 mg/100g in T_4_), calcium (from 9.66 mg/100g in T_0_ to 64.03 mg/100g in T_4_), zinc (from 0.055 mg/100g in T_0_ to 0.65 mg/100g in T_4_), and iron (from 0.64 mg/100g in T_0_ to 1.43 mg/100g in T_4_) in quince fruit leather with increasing almond powder concentrations. In quince fruit leather, the addition of almond powder enhanced overall the mineral contents, as almonds are a good source of minerals, and the addition exhibited synergistic effect that resulted in higher mineral contents in the final product (T_4_). Similar results were also observed from a study in which cookies were developed with supplemented almond flour and the obtained results in magnesium contents from 58.96 ± 0.01 to 77.16 ± 0.01 mg/100g and calcium contents increased from 185.77 ± 0.00 to 230.16 ± 0.01 [32]. Similarly, the same trend also observed in a study where soy slurry was incorporated into papaya pulp to enhanced the physicochemical characteristics of papaya leather. It was observed that the gradually increased in soy slurry also increased the Calcium and iron contents from 3.96 ± 1.72 to 32.58 ± 1.55 and 0.73 ± 0.04 to 2.45 ± 0.18, respectively [31].

**Table 3:** Effect of almond powder concentration on the mineral contents of quince leather.

Treatments	Mg (mg/100g)	Ca (mg/100g)	Zn (mg/100g)	Fe (mg/100g)
**T**_**0**_	6.667 ± 1.52^e^	9.667 ± 1.52^e^	0.056 ± 0.01^d^	0.643 ± 0.05^d^
**T**_**1**_	20.034 ± 1.51^d^	21.6 ± 1.65^d^	0.264 ± 0.08 cd	0.863 ± 0.12 ^cd^
**T**_**2**_	32.434 ± 1.80^c^	34.544 ± 1.85^c^	0.346 ± 0.09^bc^	1.014 ± 0.01^bc^
**T**_**3**_	46.933 ± 2.96^b^	47.516 ± 1.37^b^	0.501 ± 0.09^ab^	1.229 ± 0.07^ab^
**T**_**4**_	60.7 ± 1.37^a^	64.033 ± 2.17^a^	0.656 ± 0.07^a^	1.434 ± 0.11^a^

### Phytochemical analysis of protein fortified quince leather

#### DPPH (%).

Table 4 showed the DPPH radical scavenging activity of the quince fruit leathers with different levels of almond powder. The incorporation of almond powder significantly increased the DPPH contents of quince fruit leather. In this study T_4_ showed highest DPPH contents (71.533%) as compared to T_0_ (58.29%) because almond are naturally rich in Vit-E which has an ability to neutralize the free radicals. Almond also possess a good amount of phenolic and flavonoid compounds which boost up the antioxidant contents in quince fruit leather. Therefore, almond powder is synergistic with quince to enhance the antioxidant potential of quince fruit leather. The same rising pattern was observed in the AS20 samples (treatment consisting of 20% almond skin) which exhibited the maximum antioxidant effect in wheat biscuits than the control [1].

**Table 4:** Effect of almond powder concentration on the physicochemical analysis of quince fruit leather.

Treatments	DPPH (%)	TPC (mg GAE/g)	TFC (mg QE/g)
**T**_**0**_	58.294 ± 0.70^d^	1.64.75^d^ ± 0.56^d^	82.91^d^ ± 0.34^d^
**T**_**1**_	66.12 ± 0.66^c^	167.19 ± 0.84 cd	84.17 ± 0.96^c^
**T**_**2**_	67.95 ± 0.51^b^	168.20 ± 0.39^bc^	87.82 ± 0.56^bc^
**T**_**3**_	69.27 ± 0.50^b^	169.29 ± 0.45^ab^	89.10 ± 0.99^b^
**T**_**4**_	71.53 ± 0.57_a_	170.32 ± 0.55^a^	90.38 ± 0.45^a^

#### Total phenolic contents (TPC).

The TPC of quince fruit leathers prepared by varying the composition of almond powder is shown in Table 4. The incorporation of almond powder significantly increased the TPC contents of quince fruit leather. In this study T_4_ showed highest TPC contents (170.32 mg GAE/g) as compared to T_0_ (164.75 mg GAE/g) because almond naturally contained polyunsaturated fatty acids which boost up the phenolic contents of quince fruit leather [37]. A study indicated a same trend in which almond powder was used in different ratio (0–30%) and the obtained results were 174.3–314.5 mg GAE/g [38].

#### Total flavonoid contents (TFC).

Table 4 gives the total flavonoid content of quince fruit leathers fortified with almond. As the concentration of almond powder was raised, the TFC rose tremendously. T4 had the greatest TFC (90.38 mg QE/g) and T_0_ had the lowest (82.91 mg QE/g). Flavonoids, like catechin, flavanol, flavanone, and anthocyanins, are naturally present in almonds, and they all combine to increase the flavonoid content of quince fruit leather. The same trend was found in cookies made using almond and pawpaw flours with TFC values of between 1.04 and 2.98 mg QE/g [39].

### Vitamin C

**Fig 2:**
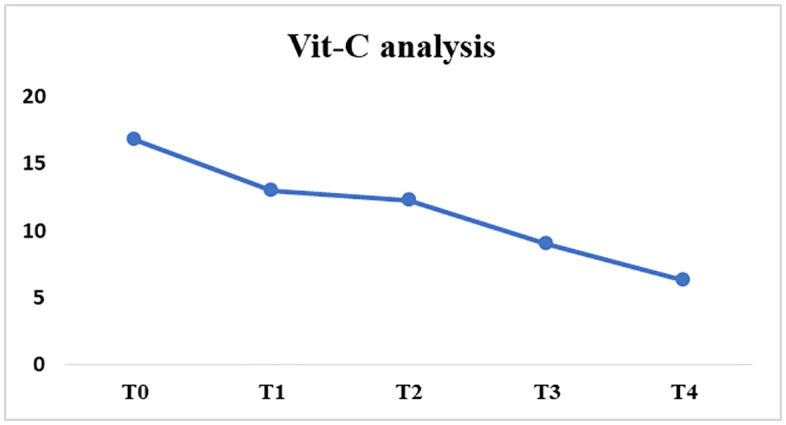
Influence of concentration of almond powder on the vitamin-C contents of quince leather.

Fig 2 presents vitamin C content of almond fortified quince fruit leather. The incorporation of almond powder significantly decreased the Vitamin-C contents of quince fruit leather. T_4_ showed lowest Vit-C (6.33 mg/100g) as compared to T_0_ (16.77 mg/100g). This loss can be explained by no vitamin C is present in almonds and the presence of high level fat in almond powder that can make ascorbic acid unstable during processing. Vitamin C is quite sensitive to heat, oxygen and light thus is very likely to be degraded during the processing of food. Through the thermal treatment and drying process that fruit leather goes through, the process can greatly stimulate its loss [40]. Moreover, the addition of almond powder can also lead to the dilution effect, which decreases the total level of vitamin C in the end product. In a previous study fruit leather was prepared by using guava fruit, beetroot powder and almond powder. The ascorbic content was decreased (225.90–220.95 mg/100g) due to fortification of beetroot powder and almond powder [41]. Similarly decreasing trend of Vitamin C was observed in almond and oat meal fortified beverage [42].

### Texture analysis

**Fig 3:**
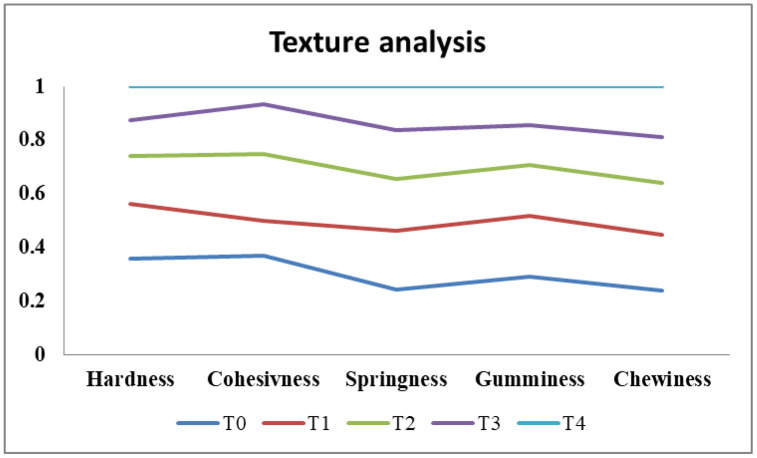
Influence of concentration of almond powder on quince leather texture.

The texture profile parameters of quince fruit leathers fortified with almond are shown in Fig 3. The addition of almond powder significantly decreased the texture (hardness, Cohesiveness, Springiness, Chewiness, and Gumminess**)** of quince fruit leather. In this study, a gradually decreased values were observed in hardness (from 64.933 in T_0_ to 22.876 in T_4_), Cohesiveness (from 1.15 in T_0_ to 0.804 in T_4_), Springiness (from 0.874 in T_0_ to 0.58 in T_4_), Chewiness (from in 66.75 in T_0_ to 52.88 in T_4_), Gumminess (from 57.83 in T_0_ to 28.58 in T_4_) because the addition of fine almond particles to quince pulp which disrupts the connectivity of the pectin-based network and weakens the contact between the particles of the fruit, leading to loss of cohesiveness and firmness. Since almonds are a rich source of fat and protein, their addition may have raised the concentration of these substances in the matrix, which are mostly engaged in the emulsification process. Also, the proteins and lipids in almond powder can interfere with the process of water retention and serve as lubricants in the matrix, weakening the structure and reducing its deformation resistance [43]. In general, the structural integrity of the pectin gel network is affected by the addition of almond powder, and this is the cause of the hardness, springiness, chewiness, and gumminess reductions observed.

The same trend was also observed in a previous study in which gluten free chips were prepared by using almond powder which showed decreasing values of hardness [44]. Reductions in cohesiveness (0.49 to 0.34) were also observed when almond flour was used instead of animal fat in beef patties [45].The same trend was also observed from previous study in which hydrocolloids was used with dragon fruit to prepare fruit leather and hydrocolloids significantly decrease the on springiness and gumminess values [46]. These finding are supported by a study in which gluten-free cupcakes were developed with the supplementation of almond flour and coconut flour [47]. Another study in which biscuits were developed by using jack fruit flour supplemented with Indian almond powder and the obtained result was a decreasing affect in gumminess [48].

### Tensile strength

**Fig 4:**
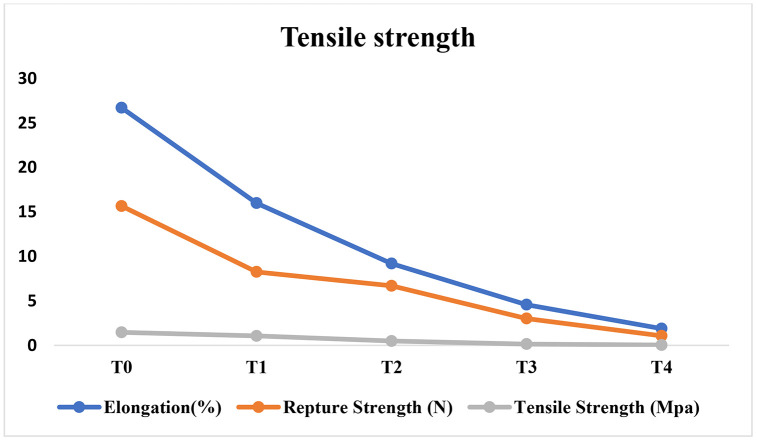
Effect of almond powder concentration on the rupture strength of the quince leather.

The addition of almond powder significantly decreased the tensile strength, elongation and rupture strength as shown in Fig 4. Tensile strength (from 1.46 MPa in T_0_ to 0.04 MPa in T_4_), elongation (from 15.66% (T_0_) to 1.067% (T_4_), rupture strength (from 64.933N (T_0_) to 22.876N (T_4_) were gradually decreased in this study. These small particles of almond disrupt the creation of a strong gel network and decrease the strength of intermolecular interactions. Moreover, the almond powder contains proteins and lipids that can be considered internal lubricants, which only decrease tensile strength and elasticity. Generally, the addition of almond powder changes the mechanical integrity of pectin network which leads to lower tensile strength, elongation and rupture strength. The tensile strength of quince fruit leather was weakened by the high fiber content of almond powder which broke the structural network. Also, the reduction in the pectin level helped in reduction of tensile strength. The same trends were observed with mango fruit leather study, in which the various plasticizers were used and tensile strength was greater in the control group than treated group because the surface tension between the particles and formation of free space [49]. Similar findings were obtained with mulberry fruit leather whereby the addition of whole wheat flour and wheat bran to mulberry pulp led to a reduction in elongation from 64.22- to 24.72, respectively [50]. Similarly, the control treatment of fruit leather made of mango fruits exhibited higher rupture strength compared to plasticizer treatments, which is due to low particle surface tension and high free volume [49].

### Color analysis

**Fig 5:**
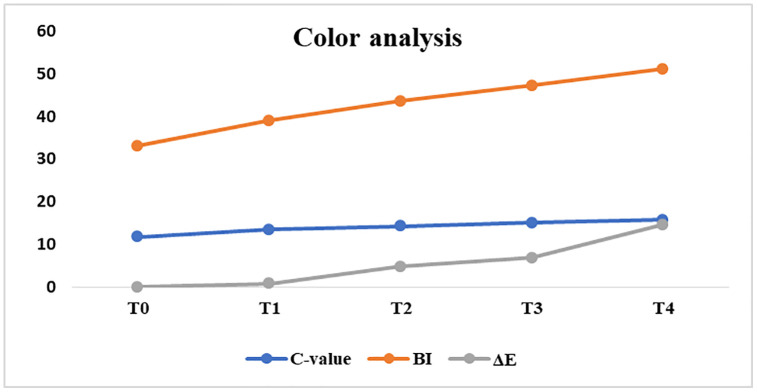
Influence of almond powder on the color of quince fruit leather.

The addition of almond powder significantly increased the color parameters as shown in Fig 5. This study showed that there was a gradual increase in color difference (ΔE) (from 0 in T_0_ to 14.64 in T_4_), chroma value (from 11.72 in T_0_ to 15.09 in T_4_), and browning index (from 33.89 in T_0_ to 51.19 in T_4_). These color changes may be explained by the fact that the almond powder contains high proportions of protein that favored the Maillard reactions and gave the quince fruit leather a darker color. The same was reported in testing the physicochemical properties of fruit leather with different types of gums used to assess the total change in color, in which the range of values between 0–5.39 were reported [51]. The same trend was observed in a previous study in which gum arabic and carrageenan were used in the development of guava-banana leather and the observed that gum arabic (13.93–16.60) had a higher chroma values in the production of fruit leather than kappa-carrageenan (12.76–15.76), which showed deep red-purple color [52]. The same result was also analyzed when the different types of gums were used to determine the physico-chemical properties of fruit leather and the observed browning index showed increasing effect within the range of 11.03–15.34 [51]. Similarly, another study showed that when apple leathers were prepared by using walnut flour then the observed browning index was increased between 30–48 and it was concluded that Oxidation and other non-enzymatic reactions may be the cause of the increasing effect. The phenolic substances presented in walnut flour and apple juice was responsible for the increase in browning index [53].

### Oil absorption capacity (OAC) and water absorption capacity (WAC)

**Fig 6:**
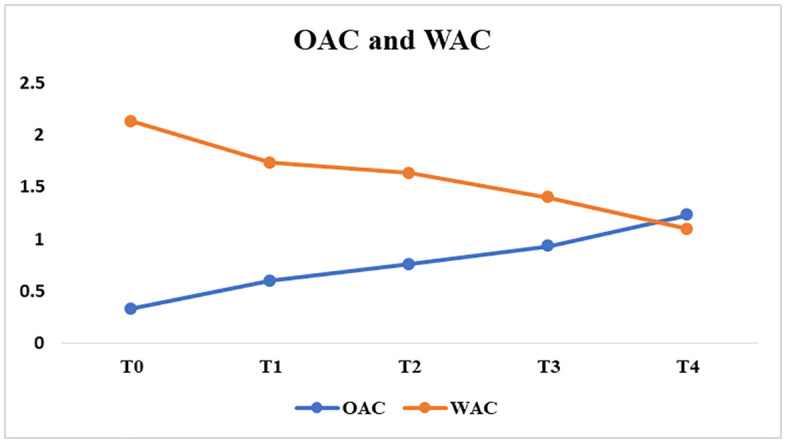
Influences of the concentration of almond powder on the WAC and OAC of the quince leather.

The oil and water absorption capacity of quince fruit leathers prepared by varying the composition of almond powder is shown in Fig 6. The addition of almond powder significantly increased the oil absorption capacity and decreased the water absorption capacity. In this study, a gradually increased in OAC was observed from 0.333 (T_0_) to 1.233 (T_4_). This is explained by the fact that almond powder contains a high level of fat that has provided the fruit leather with a porous structure that allows increased absorption of oil. Also, almond powder has a high content of protein which led to increased absorption of oil. The same trend was observed in which oat flour was used with annona pulp to prepare fruit leather where the capacity to absorb oil rose by 25.20 to 26.30 because oat flour has higher amounts of fat than annona fruit [11]. While, a gradually decreasing trend was observed in water absorption capacity (from 2.133 in T_0_ to 1.1 in T_4_). This decreasing values is probably attributed to the capacity of the almond powder to decrease the retention of water since their fat and protein factors are the hindrances to the absorption of water. The addition of almond powder was another element that helped to increase the shelf life of quince fruit leather. The same was also found in a study when wheat, almond and pawpaw fruit flours were mixed in various proportions; the mixture with 40 percent of almond flour gave the lowest WAC (2.60 to 1.15). This was explained by the lower content of carbohydrates that led to the reduction in water absorption [54].

### Physicochemical analysis of protein fortified quince leather

The physicochemical analysis of quince fruit leathers prepared by varying the composition of almond powder is shown in Table 5. The addition of almond powder significantly increased the pH and decreased the TSS and acidity. In this study, the pH was also raised gradually in T0 (3.836) to T4 (4.553) probably because of the increased pH and buffering strength of almond powder that decreased the overall acidity of the quince fruit leather. The same increasing pH trend was observed in fiber-based fruit leather made of red dragon fruit peel and mango [10]. While, a gradually decreasing trend was observed in TSS from (5.366 in T_0_ to 2.9 in T_4_). This could be explained by the fact that almond powder has less carbohydrates and the fact that its fiber amount and protein can bind soluble sugars. The same findings were described regarding persimmon fruit leather, with the TSS decreasing with the introduction of protein-rich ingredients [55]. Also, a gradually decreasing trend was observed in acidity from (0.054 in T_0_ to 0.034 in T_4_), it was due to the low level of carbohydrates which helped to diluted the natural sugar of quince fruit leather. The trend is consistent with the results of on papaya and guava mixed fruit leathers that also showed lowered acidity (73.58 to 71.66%) as more additives were added [56].

**Table 5:** Effect of almond powder concentration on the physicochemical analysis of quince fruit leather.

Treatments	pH	TSS (° brix)	Acidity (%)
**T**_**0**_	3.8367 ± 0.080^d^	5.367 ± 0.15^a^	0.054 ± 0.0015^a^
**T**_**1**_	4.3034 ± 0.015^c^	4.834 ± 0.05^ab^	0.043 ± 0.0017^b^
**T**_**2**_	4.3534 ± 0.035^bc^	3.867 ± 0.05^bc^	0.041 ± 0.0015^c^
**T**_**3**_	4.4234 ± 0.025^b^	3.534 ± 0.05^c^	0.035 ± 0.001^d^
**T**_**4**_	4.5534 ± 0.020^a^	2.901 ± 0.1^d^	0.034 ± 0.002^e^

### Sensory analysis of protein fortified quince leather

**Fig 7:**
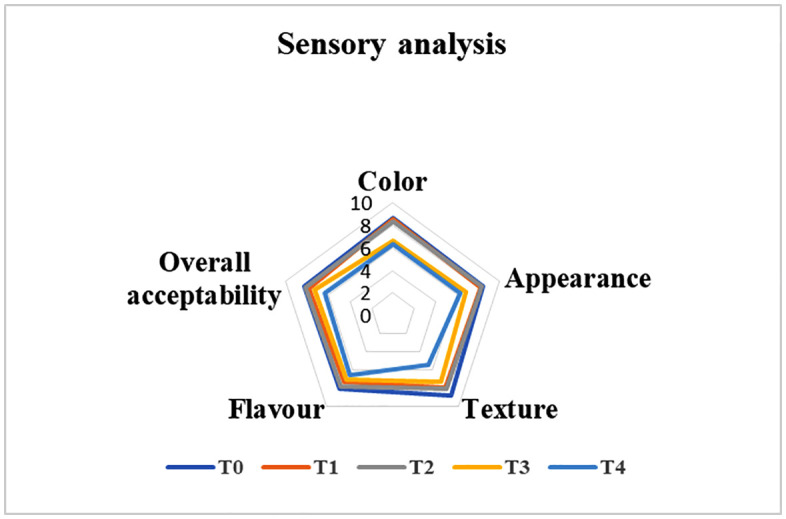
Influence of concentration of almond powder on Sensory analysis of quince fruit leather.

Quince fruit leather with 10% almond powder (T_2_) was the most acceptable of all of the treatments (Fig 7). T_2_ was smooth, soft, not sticky, and has good elasticity and firmness as explained by the panelists. This enhancement of T_2_ sensoriality could be explained by the balanced combination of quince pulp and almond powder that did not affect the flavor and mouthfeel negatively but provided more texture. Conversely, the almond higher concentrations (T_3_ and T_4_) scored relatively lower on sensory, probably because of the lipid content and particle interference, which can cause discontinuity in the structural matrix, thus producing undesirable texture and mouthfeel. The same result was reported a sensory rating of almond paste made using almond cover was between 8.89–5.05 with moderate levels of almond being more acceptable [57]. A study was conducted to analyzed the effect of almond powder, in which different protein sources were used to prepared a date bars, then the obtained result of the treatment which has 20% almond had lower flavor than 20% skim milk powder which were 7.40 and 7.70, respectively [58]. The same result also obtained from the study, in which almond based milk was used to prepared to used replaced the dairy products [59]. In another study almond milk was used to prepared probiotic yogurt and almond showed a significant effect on rating appearance of product (5–3.0) [60]. Other products have also been noted to have the impact of almond powder on texture. The gluten-free chips that are made using almond powder (30 percent) were found to be less hard, whereas the cakes made using 30 percent almond powder demonstrated better overall acceptability compared to the 50–70 percent almond powder treatment [44,61].These findings corroborate the fact that the moderate use of almond powder can be used to improve sensory characteristics without interfering with texture and acceptability.

## Conclusion

This research demonstrated that almond powder can successfully improve nutritional, functional and sensory properties of quince fruit leather. Protein level, fat, fiber, ash, minerals, and antioxidant activity (TPS, TFC, and DPPH) were greatly enhanced by addition of almond which indicates that almond is a promising nutritious functional snack. Although increasing the level of almond decreased the texture and tensile strength by a small margin, sensory assessment showed that the almond powder with 10% was the best formulation high sensory acceptance. The results indicate the possibility of almond-enriched quince fruit leather nutritious, healthy, sustainable snack that can be sold to interested consumers. Further studies are needed on the improved drying methods, as evidenced by vacuum or freeze drying, and on microencapsulation methods, to further improve nutrient retention, shelf stability and bioactivity of the compounds. Moreover, there is a need to study consumer acceptance, bioavailability of nutrients, and predictive shelf-life in a variety of packaging and storage conditions that will facilitate commercial production and greater use of fortified fruit products.
